# Macroeconomic dataset for comparative studies on coastal and inland regions in innovation space of Russia

**DOI:** 10.1016/j.dib.2019.104640

**Published:** 2019-10-14

**Authors:** Anna A. Mikhaylova, Andrey S. Mikhaylov, Oksana V. Savchina

**Affiliations:** a Immanuel Kant Baltic Federal University, 23016, Kaliningrad, Russian Federation; b Saint Petersburg Electrotechnical University “LETI”, 197022, Saint-Petersburg, Russian Federation; c Peoples' Friendship University of Russia, 117198, Moscow, Russian Federation

**Keywords:** Coastal region, Regional innovation system, Innovation dynamics, Innovation security, Innovation trajectory, Russia

## Abstract

This article presents regional-level data that can be used for comparative territorial studies on innovation dynamics. The dataset covers a series of 50 indicators grouped into a matrix of 5 elements of regional innovation system (human resources – HR, infrastructure, research & development sector – R&D, innovative milieu, framework conditions) and 5 components of innovation security (economic, scientific and technological – S&T, social, political, geo-ecological). This complex set of interrelated data enables to grasp the catalyst and inhibitor factors that have a significant impact on the sustainable development of a particular regional innovation system. The innovation security approach used enables to consider the locus of innovation processes, account for the relationship between individual components of regional innovation systems and acknowledge for the unique properties of the regions. The database includes statistics for a total set of 85 regions of the Russian Federation over a period of 2015 and 2016. Spatial differentiation is made on to coastal and inland regions. This enables to identify the development patterns as influenced by the global trend of coastalization.

**Table:** Specifications Table

Subject	Geography, Planning and Development
Specific subject area	Knowledge geography
Type of data	Table Figure
How data were acquired	Data are acquired from the Federal Service of State Statistics of the Russian Federation (Rosstat), Scopus database, SciVal, Scientific Research Institute – Federal Research Centre for Projects Evaluation and Consulting Services (SRI FRCEC), Single portal of the budget system of the Russian Federation (Electronic budget), Scientific and technological infrastructure of the Russian Federation – Centers for collective use of scientific equipment and unique scientific installations (Ministry of Education and Science of the Russian Federation), Association of Accelerators and Business Incubators of Russia, Information and communication support system for young innovators (ICS)
Data format	Raw Analyzed Filtered
Parameters for data collection	Data is structured by merging information from the aforementioned sources. Sample construction involved conversion of raw data collected from the various sources into indicators and coefficients of a comparable form. Extrapolation is applied for periods where data were not available. Data is aggregated by elements of regional innovation system and components of innovation security. Innovation security matrices are calculated for all regions of the Russian Federation. The ranking approach is applied to all regions according to the level of innovation security, identifying the strengths and weaknesses of their regional innovation systems. A comparative analysis of the innovation security of coastal and inland regions of the Russian Federation is undertaken.
Description of data collection	Presented data covers a series of regional-level data on the most important indicators used in socio-economic and geo-economic research in conducting a comparative assessment of the level of regional innovation development and innovation security.
Data source location	Central Federal District – 18 regions of the Russian Federation: Belgorod region, Bryansk region, Vladimir region, Voronezh region, Ivanovo region, Kaluga region, Kostroma region, Kursk region, Lipetsk region, Moscow region, Orel region, Ryazan region, Smolensk region, Tambov region, Tver region, Tula region, Yaroslavl region, Moscow – city of federal importance. Northwestern Federal District – 11 regions of the Russian Federation (additionally presented aggregated data for the Arkhangelsk region with the inclusion of the autonomous region): Republic of Karelia, Komi Republic, Arkhangelsk region, Nenets Autonomous Area, Vologda region, Kaliningrad region, Leningrad region, Murmansk region, Novgorod region, Pskov region, St. Petersburg – city of federal importance. Southern Federal District – 8 regions of the Russian Federation: Republic of Adygeya, Republic of Kalmykia, Republic of Krym, Krasnodar Territory, Astrakhan region, Volgograd region, Rostov region, Sevastopol – city of federal importance. North Caucasus Federal District – 7 regions of the Russian Federation: Republic of Daghestan, Republic of Ingushetia, Kabardino-Balkarian Republic, Karachayevo-Circassian Republic, Republic of North Ossetia – Alania, Chechen Republic, Stavropol Territory. Volga Federal District – 14 regions of the Russian Federation: Republic of Bashkortostan, Republic of Mari El, Republic of Mordovia, Republic of Tatarstan, Udmurtian Republic, Chuvash Republic, Perm Territory, Kirov region, Nizhny Novgorod region, Orenburg region, Penza region, Samara region, Saratov region, Ulyanovsk region. Ural Federal District – 6 regions of the Russian Federation (additionally presents aggregated data for the Tyumen region with the inclusion of autonomous districts): Kurgan region, Sverdlovsk region, Khanty-Mansi Autonomous Area – Yugra, Yamal-Nenets Autonomous Area, Tyumen region, Chelyabinsk region. Siberian Federal District – 12 regions of the Russian Federation: Republic of Altai, Republic of Buryatia, Republic of Tuva, Republic of Khakassia, Altai Territory, Trans-Baikal Territory, Krasnoyarsk Territory, Irkutsk region, Kemerovo region, Novosibirsk region, Omsk region, Tomsk region. Far Eastern Federal District – 9 regions of the Russian Federation: Republic of Sakha (Yakutia), Kamchatka Territory, Primorye Territory, Khabarovsk Territory, Amur region, Magadan region, Sakhalin region, Jewish Autonomous region, Chukotka Autonomous Area.
Data accessibility	With the article

**Table:** 

**Value of the Data** • Traditional approach to the evaluation of regional innovation development implies consideration of a narrow range of indicators generally focused on determining the economic competitiveness, innovation infrastructure, and scientific productivity or R&D expenditure [[1], [2], [3], [4], [5], [6], [7], [8], [9], [10], [11]]. To a large extent, this factor predetermines the research results with a ranking table invariably led by core regions – the major financial and industrial centres. These results rarely differ from general assessments of socio-economic development [[12], [13], [14], [15], [16], [17], [18]]. The database presented addresses such research limitations by taking into account the full range of factors affecting the innovation development of regions and their innovation security. As a result, a different picture of the national innovation system is obtained featuring heterogeneity of the innovation space given the broad scope of indicators evaluated. • The dataset covers a series of 50 indicators for a total set of 85 regions of the Russian Federation that is structured in a regional innovation security evaluation matrix [[19], [20], [21]]. The wide spectrum of parameters used ensures a comprehensive assessment of each of the 5 innovation security components (economic, scientific and technological, social, political, geoecological) being interrelated to the values of regional innovation system elements (human resources, infrastructure, R&D, innovative milieu, framework conditions). The data provided enables regional scientists to conduct comparative studies using individual criteria selected, including the assessment of the region's position relative to average values for federal districts or nationwide. Of particular value would be research on the typologies of regions regarding their geo-economic position – coastal and inland, borderland and midland, central and peripheral, etc. • This dataset may have important policy implications. The detailed perspective over the regional innovation divergence enabled to isolate gaps that threaten the innovation security of a region and inhibit the development of its innovation system. Correlations may be found between certain policy instruments implemented and the change in macroeconomic indicators. The data may be applied to the elaboration of a territorial-adaptive approach to regional development taking into account the differences in the territorial capital of regions.

## 1 Data

The data cover a sample of 85 regions of the Russian Federation, of which 23 are coastal regions and 62 inland regions. The coverage data period is 2015–2016. The data are grouped: 1) by components of innovation security: economic, scientific and technological – S&T, social, political, geoecological; 2) by elements of regional innovation system: human resources – HR, infrastructure, research & development sector – R&D, innovative milieu, framework conditions); and 3) by types of regions, selected on the basis of their economic and geographical position: coastal, inland.

Innovation security assessed using 50 indicators. A number of factors determine the choice of indicators. Firstly, the comprehensiveness of the research – the need to evaluate all components of innovation security: economic, S&T, social, political, geoecological in their relationship with the components of RIS: HR, infrastructure, R&D sector, innovative milieu, framework conditions. Secondly, the implementation of the principle of sufficiency – 2 major indicators are used to assess each component of innovation security in each of 5 aspects (HR, infrastructure, R&D sector, innovative milieu, framework conditions) characterizing the development of regional innovation system (RIS) components. Thirdly, the availability of dynamic data series for all subjects of the Russian Federation in order to conduct annual monitoring of innovation security.

Supplement 1 presents the typology of Russian regions to coastal and inland types.

Supplements 2–9 present the matrix of innovation security for all administrative units of the Russian Federation for 2015, 2016.

The original series of aggregated macroeconomic data for innovation security matrices are available in separate Excel spreadsheets (Supplements 10, 11).

Fig. 1, Fig. 2, Fig. 3, Fig. 4, Fig. 5, Fig. 6, Fig. 7, Fig. 8, Fig. 9, Fig. 10 are presenting the development of regional innovation system elements across regions of the Russian Federation in 2015–2016, indicating RIS elements that excel national average values. Coastal regions are marked with blue filling.

**Fig. 1:**
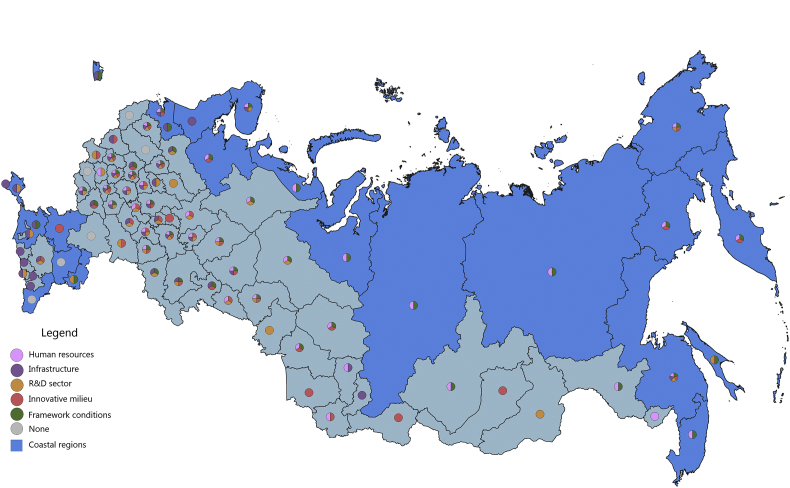
The economic component of the regional innovation security, 2015.

**Fig. 2:**
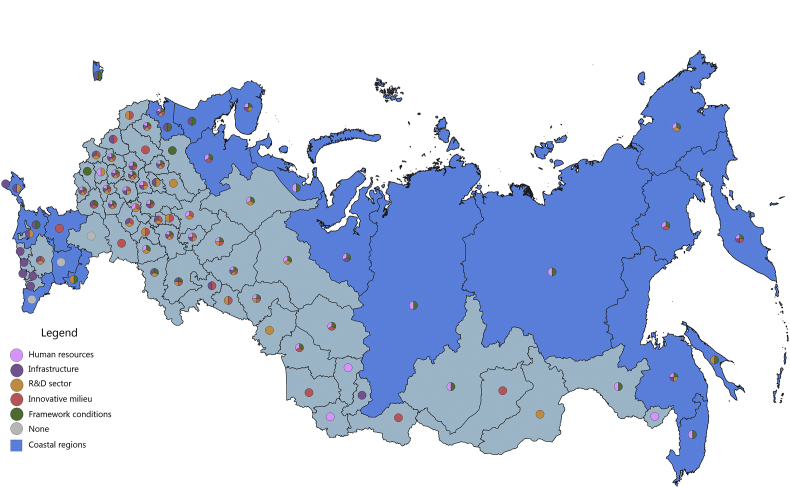
The economic component of the regional innovation security, 2016.

**Fig. 3:**
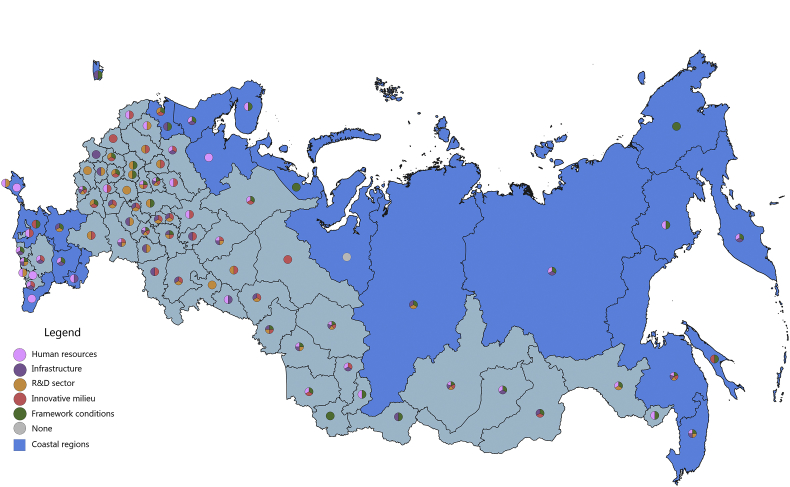
The scientific and technological component of the regional innovation security, 2015.

**Fig. 4:**
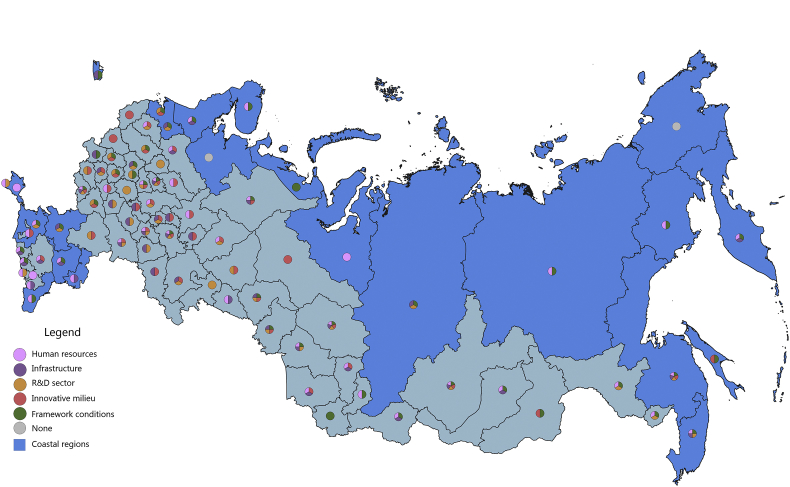
The scientific and technological component of the regional innovation security, 2016.

**Fig. 5:**
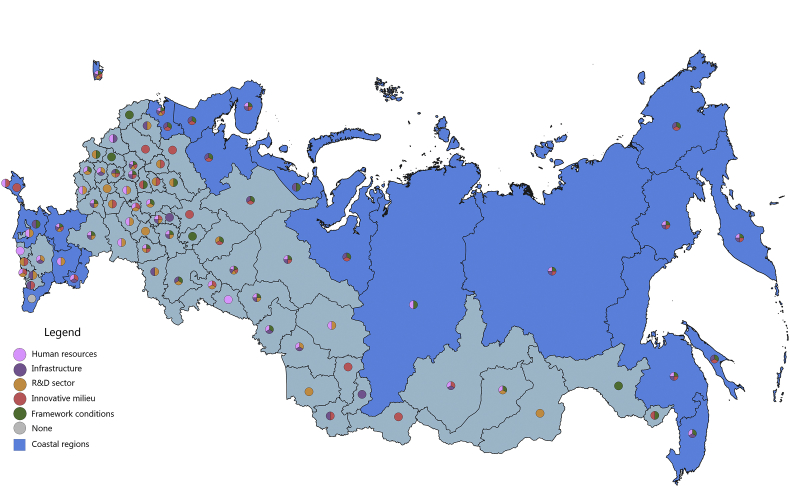
The social component of the regional innovation security, 2015.

**Fig. 6:**
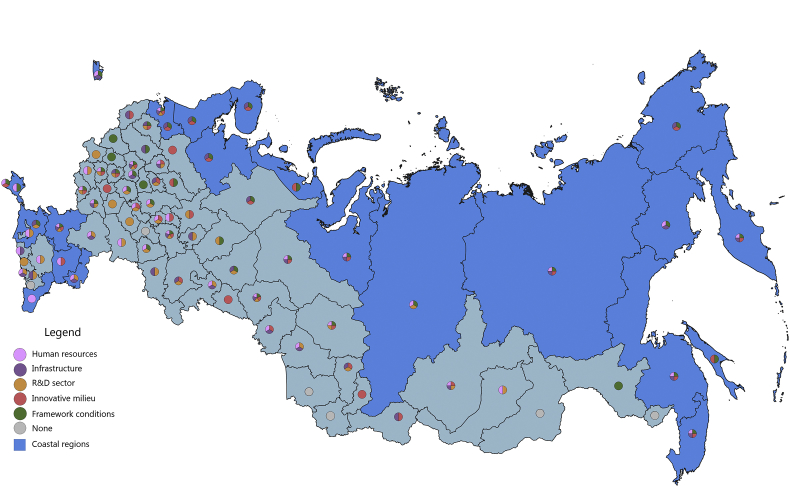
The social component of the regional innovation security, 2016.

**Fig. 7:**
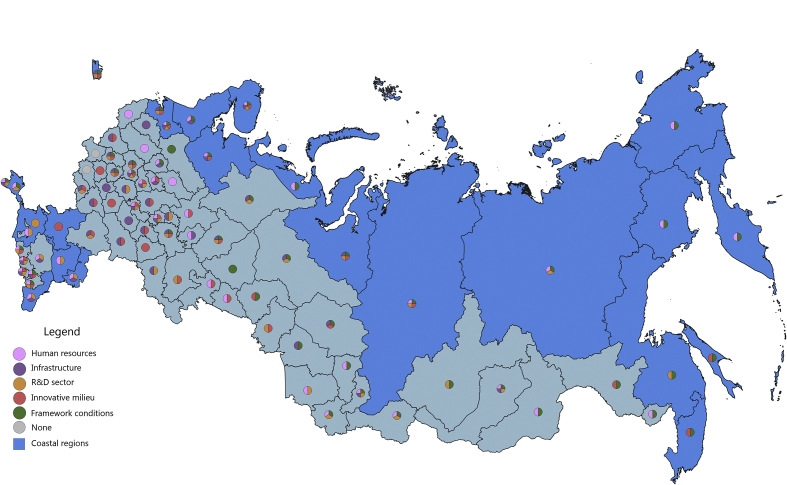
The political component of the regional innovation security, 2015.

**Fig. 8:**
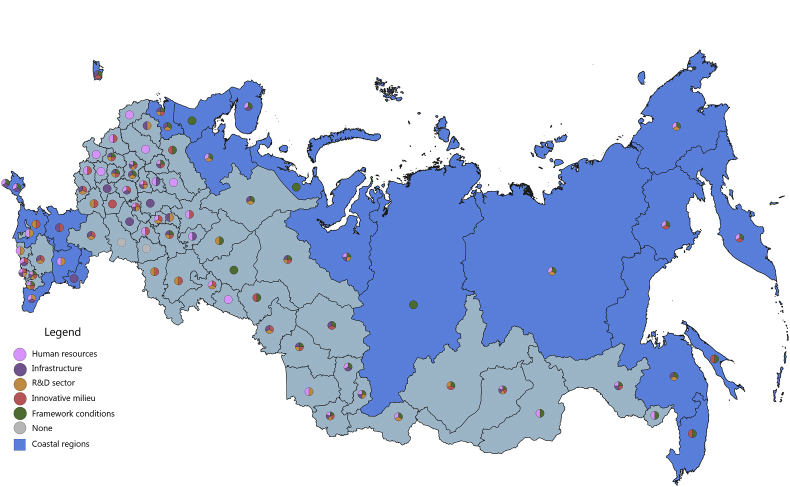
The political component of the regional innovation security, 2016.

**Fig. 9:**
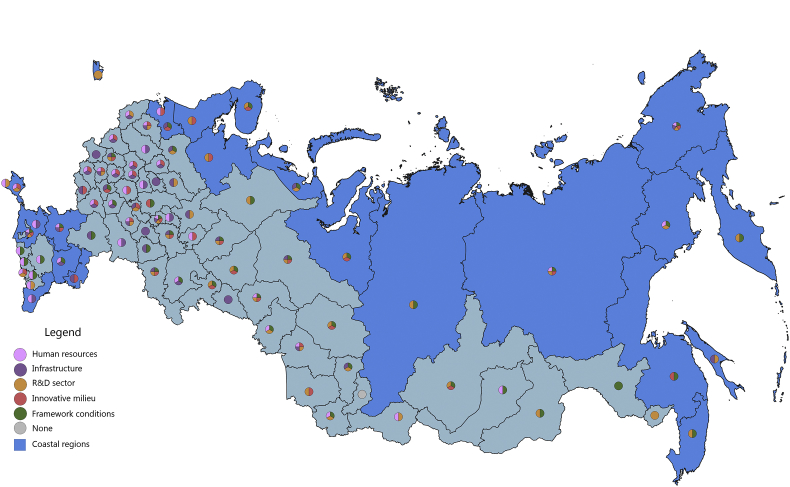
The geoecological component of the regional innovation security, 2015.

**Fig. 10:**
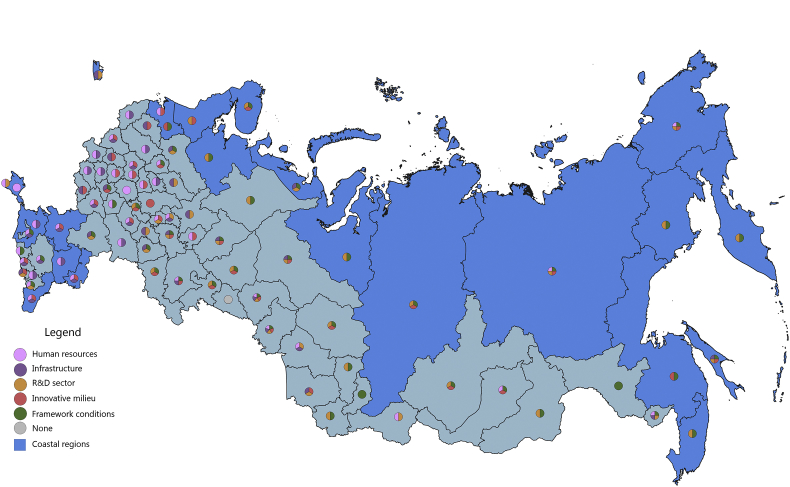
The geoecological component of the regional innovation security, 2016.

**Fig. 11:**
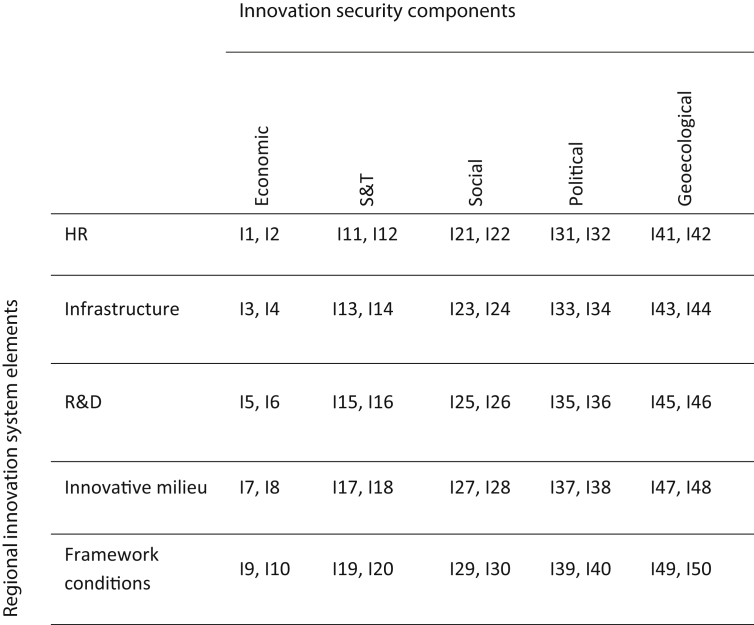
Regional innovation security evaluation matrix. Note. I – indicator; 1, 2, 3 … 50 – indicator number.

Fig. 11 features an evaluation matrix applied for measuring the level of regional innovation security.

Table 1, Table 2, Table 3, Table 4, Table 5 contain detailed data on indicators and evaluation procedure for assessing the components of the innovation security of a region.

**Table 1:** Indicators for assessing the economic component of the innovation security of a region.

Indicator title and its position in the matrix	Indicator calculation	Data source/frequency
I1 – The costs of organizations for the education and training of human resources related to innovation, per 1 employed in the economy, rubles per person.	Calculated as the ratio of the costs of organizations on the education and training of human resources associated with innovation to the average annual number of employees Republic of Krym and Sevastopol for 2015 - n/a (equated to 0)	Rosstat/Annual
I2 – The ratio of high-performance workplaces to the average annual number of employees, units per person.	Calculated as the ratio of the number of high-performance jobs to the average annual number of employees	Rosstat/Annual
I3 – The share of organizations with websites, %	Raw data	Rosstat/Annual
I4 – Density of public roads with hard surface, km of tracks per 1000 km^2^ of territory	Raw data	Rosstat/Annual
I5 – The proportion of expenditure on research and development aimed at the development of the economy, in the total domestic expenditure on research and development, %	Raw data	Rosstat/Annual
I6 - The costs of organizations for patents, licenses for the use of inventions, industrial designs, utility models rub. per 1000 rubles of product innovation shipped	Calculated as the ratio of the costs of organizations for patents, licenses for the use of inventions, industrial designs, utility models to the volume of innovative products delivered	Rosstat/Annual
I7 – The share of high-tech and knowledge-intensive industries in the Gross Regional Product (GRP), %	Raw data	Rosstat/Annual
I8 – Innovative activity of organizations, %	Raw data	Rosstat/Annual
I9 – GRP per capita, thousand rubles per person	Raw data	Rosstat/Annual
I10 – Investments in fixed capital per capita, rubles per person	Raw data	Rosstat/Annual

**Table 2:** Indicators for assessing the scientific and technological component of the innovation security of a region.

Indicator title and its position in the matrix	Indicator calculation	Data source/frequency
I11 – The share of researchers with a scientific degree of candidate and Doctor of Science in the total number of human resources engaged in research and development, %	Calculated as the ratio of the number of researchers with a scientific degree of candidate and Doctor of Sciences to the total number of human resources engaged in research and development. Data on the number of researchers and human resources engaged in research and development for 2015 and 2016 for the Jewish Autonomous Region are not available (confidential). Therefore, in the Jewish Autonomous Region, 2015 was replaced by 2012, and 2016 – by 2013.	Rosstat/Annual
I12 - Salary in the field of research and development to the regional average, times	Raw data For Nenets Autonomous Area data for 2015 and 2016 is unavailable; it is replaced by the average for the Arkhangelsk region, including the Nenets Autonomous Area. For the Jewish Autonomous Region data for 2015, 2016 is unavailable; it is replaced by the average in the Far Eastern Federal District, which includes the Jewish Autonomous Region. For the Republic of Ingushetia, data for 2015 are not available, therefore in 2015 data for 2016 are indicated.	Rosstat/Annual
I13 – The number of organizations of innovation and specialized infrastructure per 1000 organizations performing research and development	Calculated as the ratio of the number of organizations of innovative and specialized infrastructure to the number of organizations performing research and development. The organizations of innovation and specialized infrastructure include centers for collective use, fab labs, engineering centers, innovation centers, prototyping centers, and certification centers.	Ministry of Education and Science of the Russian Federation, Association of Accelerators and Business Incubators of Russia, ICS, Rosstat/Annual
I14 – Number of small innovative enterprises per 1000 researchers	Calculated as the ratio of the number of operating small innovative enterprises to the number of researchers. Only small innovative enterprises included in the register of the SRI FRCEC are counted.	SRI FRCEC, Rosstat/Annual
I15 – Domestic costs of research and development from GRP, %	Raw data	Rosstat/Annual
I16 – The number of patents for inventions issued by Rospatent to Russian applicants per 1 million people of population	Raw data	Rosstat/Annual
I17 – The share of extra-budgetary funds in domestic expenditure on research and development, %	Raw data	Rosstat/Annual
I18 – Excess in the volume of exports of technology and services of a technical nature over imports, times	Calculated as the ratio of the export of technologies and services of a technical nature to the import of technologies and services of a technical nature. For the Ivanovo region, data for 2016 is not available, it is replaced by 2015. For the Magadan region, data for 2015 is not available, it is replaced with the average for the Far Eastern Federal District. For Chuvash Republic, data for 2015 is not available, it is replaced with the average for the Volga Federal District. For the Republic of Sakha (Yakutia) data for 2015, 2016 is inaccessible (confidential), it is replaced by the average in the Far Eastern Federal District.	Rosstat/Annual
I19 – The degree of depreciation of fixed assets in a full range of organizations at the end of the year, %	Raw data	Rosstat/Annual
I20 – The number of scientific articles published in the Scopus database per 1000 people, average annual population	Calculated as the ratio of the number of scientific articles published in the Scopus database to the average annual population. Data is sourced by city and institutional affiliation for 2015, 2016.	Scopus (SciVal), Rosstat/Annual

**Table 3:** Indicators for assessing the social component of the innovation security of a region.

Indicator title and its position in the matrix	Indicator calculation	Data source/frequency
I21 – Percentage of university students per 10,000 people, pers.	Raw data	Rosstat/Annual
I22 – The share of the employed population with higher professional education at the age of 25–64, %	Raw data	Rosstat/Annual
I23 – The share of households with access to the Internet, %	Raw data	Rosstat/Annual
I24 – The number of subscriber devices of mobile radio telephone (cellular) communication per 1000 people of population	Raw data For the Moscow region, data for 2015, 2016 is inaccessible (confidential), it is replaced by data for Moscow. For Nenets Autonomous Area data for 2015, 2016 is unavailable; it is replaced by the average for the Arkhangelsk region, including the Nenets Autonomous Area. For the Leningrad region, data for 2015, 2016 is inaccessible (confidential); it is replaced with data on St. Petersburg. For Sevastopol, the data for 2015 is not available; it is replaced with data for 2016.	Rosstat/Annual
I25 – Postgraduate students with the defense of a doctoral (PhD) thesis per organization dedicated to postgraduate training, people	Calculated as the ratio of the number of postgraduate students (PhD) with thesis defended to the number of organizations involved in postgraduate teaching.	Rosstat/Annual
I26 – Postgraduate and doctoral students with thesis defense per 10,000 people population	Calculated as the ratio of the sum of the number of postgraduate (PhD) students with the dissertation defense and the number of doctoral (Habilitation) students with the dissertation defense to the average annual population.	Rosstat/Annual
I27 – The share of the population that used the Internet to order goods and (or) services in the total population, %	Raw data	Rosstat/Annual
I28 – Percentage of population not using the Internet for security reasons, %	Raw data	Rosstat/Annual
I29 – Average per capita income of the population, rubles per person	Raw data	Rosstat/Annual
I30 – The demand of organizations for employees to fill vacant jobs in % of the total number of jobs for this year	Raw data	Rosstat/Annual

**Table 4:** Indicators for assessing the public component of the innovation security of a region.

Indicator title and its position in the matrix	Indicator calculation	Data source/frequency
I31 – Expenditures of the consolidated budget of the region on education from GRP, %	Calculated as the ratio of the consolidated budget of the administrative unit of the Russian Federation for education to its GRP	Electronic budget, Rosstat/Annual
I32 – The share of civil servants who have received additional vocational training outside the territory of the Russian Federation in the total number of persons who have replaced civil service positions and who have received additional vocational training	Raw data	Rosstat/Annual
I33 – The share of public authorities and local governments, which had a speed of data transmission via the Internet of at least 2 Mbit/s, %	Raw data	Rosstat/Annual
I34 – The share of electronic document circulation between state authorities, in the total volume of interdepartmental document circulation, %	Raw data; For Nizhny Novgorod region, data for 2016 is not available; it is replaced with data for 2015. For Krasnoyarsk Territory, data for 2015, 2016 is not available; it is replaced by the average value for the Siberian Federal District. For the Republic of Tyva and the Republic of Khakassia, the data for 2016 is not available; it is replaced with data for 2015.	Rosstat/Annual
I35 – The amount of expenditure on research in the total expenditures of the consolidated budget of the region (for the Russian Federation without taking into account research in the field of national defense), %	Calculated as the ratio of the volume of public expenditure on research of the administrative unit of the Russian Federation in the total expenditures of its consolidated budget	Electronic budget/Annual
I36 – The share of expenditures on scientific research of the consolidated budget of a region (for the Russian Federation without taking into account research in the field of national defense) per 1 researcher, %	Calculated as the ratio of the volume of public expenditure on research to the number of researchers.	Electronic budget, Rosstat/Annual
I37 - The volume of funds of the consolidated budget of the region, provided for the implementation of all federal targeted programs, per 1000 rubles GRP, rubles	Calculated as the ratio of the volume of funds of the consolidated budget of the administrative unit of the Russian Federation provided for the implementation of all federal targeted programs to the GRP	Rosstat/Annual
I38 – The share of the population that used the Internet to obtain state and municipal services in the total population that received state and municipal services	Raw data	Rosstat/Annual
I39 – Revenues in the consolidated budget of the region for 1 employed, thousand rubles per person	Calculated as the ratio of the amount of income in the consolidated budget of the administrative unit of the Russian Federation to the average annual number of employees of the administrative unit of the Russian Federation	Electronic budget, Rosstat/Annual
I40 – The volume of social expenses (health care, education, social policy) in the consolidated budget of the region per capita, rubles per person	Calculated as the ratio of the volume of social expenses in the consolidated budget of the administrative unit of the Russian Federation to the average annual population of the administrative unit of the Russian Federation. Social expenditures include health care, education, and social policy.	Electronic budget, Rosstat/Annual

**Table 5:** Indicators for assessing the geoecological component of the innovation security of a region.

Indicator title and its position in the matrix	Indicator calculation	Data source/frequency
I41 – The share of workers in manufacturing organizations working in hazardous and dangerous working conditions, of the total number employed in manufacturing industries, %	Raw data. For the Arkhangelsk region without the Nenets Autonomous Area data for 2015, 2016 is unavailable; it is replaced by data for the Arkhangelsk region, taking into account the Nenets Autonomous Area. For the Tyumen region without autonomous areas, the data for 2015, 2016 is not available; it is replaced with data for the Tyumen region, including autonomous areas.	Rosstat/Annual
I42 - The concentration of labor people per sq.km	Calculated as the ratio of the average annual labor force in administrative unit of the Russian Federation to the total area of the administrative unit of the Russian Federation	Rosstat/Annual
I43 – Payment for excess emissions of pollutants (disposal of production and consumption waste) per organization, thousand rubles	Calculated as the ratio of payments for excessive emissions of pollutants (disposal of production and consumption waste) to the number of enterprises and organizations of the administrative unit of the Russian Federation	Rosstat/Annual
I44 – Payment for environmental protection services per organization, thousand rubles	Calculated as the ratio of payment for environmental protection services to the number of enterprises and organizations of the administrative unit of the Russian Federation	Rosstat/Annual
I45 – The number of articles published annually in the “Environmental Sciences” section of the Scopus database per 1000 researchers	Calculated as the ratio of annually published articles of the “Environmental Sciences” subject area in the Scopus-indexed periodicals to the number of researchers. Data is sourced by city and institutional affiliation for 2015, 2016. For the Jewish Autonomous Region, data on the number of researchers for 2015, 2016 is not available (confidential); it is replaced with data for 2013, which is also taken into account in the total amount of the Far Eastern Federal District.	Scopus, Rosstat/Annual
I46 – The cost of research and development to reduce the negative anthropogenic impacts on the environment in the current costs of environmental protection for 1 person employed in the field of research and development, rubles per person	Calculated as the ratio of expenditure on research and development to reduce the negative anthropogenic environmental impacts in the current costs of environmental protection to the number of human resources engaged in research and development.	Rosstat/Annual
I47 – The share of organizations that carried out environmental innovations in the total number of organizations surveyed, %	Raw data	Rosstat/Annual
I48 - The dedicated costs associated with environmental innovation, per organization, mln rubles per unit	Raw data	Rosstat/Annual
I49 – Concentration of emissions into the atmosphere from stationary and mobile sources of pollution, tones per sq.km	Calculated as the ratio of emissions into the atmosphere of pollutants from stationary and mobile sources in the administrative unit of the Russian Federation to the area of the administrative unit of the Russian Federation	Rosstat/Annual
I50 – The share of investments aimed at protecting the environment and in environmental management from GRP, %	Calculated as the ratio of investments aimed at environmental protection and rational environmental management to the GRP of the administrative unit of the Russian Federation. Data is of 2014.	Rosstat/Biannual

The economic component of innovation security was most efficiently implemented in 2015 in 43 administrative units of the Russian Federation, of which 9 are coastal (in 2016, these are 44 regions, including 10 coastal). Thus, the value of the index of the economic component of innovation security in these regions is higher than the median value for all regions of the Russian Federation (for 2015 it is ≥0.208, for 2016 it is ≥0.224). In 2015, the top-10 included 3 coastal regions: St. Petersburg, Nenets Autonomous Area, and Chukotka Autonomous Area. In 2016, the Yamal-Nenets Autonomous Area was also added.

In 2015, the coastal regions of the Russian Federation were characterized by partial economic security of the RIS subsystems. Of the 23 coastal regions, only 7 regions have economic security indicators above national average by 3 or more RIS subsystems (Fig. 1). These regions are located on the north-western and north-eastern coasts of the Russian Federation (Khabarovsk Territory, Chukotka Autonomous Area, Murmansk region, St. Petersburg, Arkhangelsk region, Magadan region, Kamchatka Territory). Generally, the economic component of innovation security in 2015 in the coastal regions of the Russian Federation was sufficient in 1–2 RIS subsystems or was insufficient at all (Republic of Kalmykia, Republic of Daghestan). In 5 coastal regions (Primorye Territory, Krasnoyarsk Territory, Republic of Sakha (Yakutia), Nenets Autonomous Area, and Yamal-Nenets Autonomous Area) the HR component and the framework conditions of the RIS are the least economically vulnerable. In the 3 coastal regions (Kaliningrad, Leningrad Oblasts, Krasnodar Territory) these are infrastructure component and framework conditions of RIS. In the coastal regions of Astrakhan and Sakhalin – the R&D sector and the framework conditions, and in the Republic of Krym – the infrastructure and R&D components of the RIS.

In 2016, there were some changes in the innovation security systems of coastal regions compared to 2015 (Fig. 2). St. Petersburg and Kamchatka Territory strengthened their economic component of innovation security by increasing the convergence of economic development of RIS subsystems. St. Petersburg strengthened the R&D subsystem by significantly increasing the expenditure of organizations for patents, licenses for the use of inventions, industrial designs, utility models. And the Kamchatka Territory has improved the infrastructure subsystem of the RIS by increasing the digitalization of business. The Chukotka Autonomous Area and the Khabarovsk Territory, on the contrary, demonstrated a decrease in the level of innovation security in the context of the economic component due to the deterioration of the RIS innovative milieu (a significant decrease in entrepreneurial innovation activity).

The majority of coastal regions of the Russian Federation have favourable economic framework conditions for conducting innovation activities and for realizing human potential for the development of innovations. However, in general, the remaining major RIS subsystems (infrastructure, R&D, innovative milieu) have weak economic development.

The S&T component of innovation security was most efficiently implemented in 2015 in 43 administrative units of the Russian Federation, of which 12 are coastal (in 2016, these are 46 regions, including 11 coastal). Thus, the value of the index of the S&T component of innovation security in these regions is higher than the median value for all regions of the Russian Federation (for 2015 it is ≥0.258, for 2016 it is ≥0.242). In 2015, the top-10 included 2 coastal regions: St. Petersburg and Primorye Territory. In 2016, the St. Petersburg and Khabarovsk Territory, while Primorye Territory was ranked 11th place.

In 2015, the coastal regions of the Russian Federation were characterized by partial S&T security of the RIS subsystems. Of the 23 coastal regions, only 9 regions have S&T security indicators above national average by 3 or more RIS subsystems (Fig. 3). The most convergent S&T component of innovation security was represented in the Khabarovsk Territory, where all 5 RIS subsystems are involved. Primorye Territory, to a greater extent, requires attention to the S&T factor of the innovative milieu of RIS. Leningrad region is lacking S&T development of HR and infrastructure subsystems of RIS; Krasnoyarsk Territory and the Rostov region – the S&T development of HR and R&D subsystems of RIS; Kamchatka Territory, Republic of Sakha (Yakutia), Republic of Karelia, Republic of Kalmykia – to the S&T development of the R&D subsystem and innovative milieu of the RIS. RIS of 14 coastal regions of the Russian Federation turned out to be the most vulnerable in S&T terms, 8 of them involved 2 RIS subsystems, 4 – had 1 RIS subsystem in ensuring innovative security. In most of these regions, relatively favourable framework conditions have been formed and an impetus for S&T development has been developed by at least one of the RIS subsystems: HR, infrastructure or innovative milieu. However, other RIS subsystems are at risk. The threat of innovation security in the context of the S&T component was especially acute for the Yamal-Nenets Autonomous Area, where the values of all components were below the national average.

In 2016, there were some changes in the innovation security systems of the coastal regions compared to 2015 (Fig. 4). The Yamal-Nenets Autonomous Area strengthened its innovation security system through the S&T development of the HR component of RIS. Also, positive changes have occurred in the RIS of the Krasnodar Territory, the Republic of Daghestan, the Leningrad region, including due to the direct development of R&D activities. The weakening of the S&T component of innovation security was recorded in the Republic of Sakha (Yakutia), and the Chukotka Autonomous Area and the Arkhangelsk region moved to the risk zone (the level of S&T development of all their RIS subsystems in 2016 was below the national average).

The social component of innovation security was most efficiently implemented in 2015 in 43 administrative units of the Russian Federation, of which 16 are coastal (in 2016, these are 43 regions, including 20 coastal). Thus, the value of the index of the social component of innovation security in these regions is higher than the median value for all regions of the Russian Federation (for 2015 it is ≥0.346, for 2016 it is ≥0.349).

In 2015, the top-10 included 6 coastal regions: St. Petersburg, Magadan region, Yamal-Nenets Autonomous Area, Chukotka Autonomous Area, Kaliningrad region, Kamchatka Territory (Fig. 5). In 2016, top-10 regions by this indicator also included 6 coastal regions with the exception of the Magadan region, which moved to the 12th position, and its place was taken by the Murmansk region.

In general, in 2015–2016 coastal regions of the Russian Federation demonstrate a good level of social security component of their RIS. In 2015, all 5 RIS subsystems in the context of the social component of innovative security were developed in coastal regions of Rostov region, Kaliningrad region, St. Petersburg. Another 5 regions (Murmansk region, the Republic of Sakha (Yakutia), Magadan region, Kamchatka Territory, Khabarovsk Territory) have good social indicators for 4 RIS subsystems with the exception of the R&D subsystem.

For a number of regions, the social component of innovation security is represented by the development of 3 RIS subsystems (Fig. 6). The Leningrad region, the Republic of Karelia, the Arkhangelsk region, the Yamal-Nenets Autonomous Area, the Chukotka Autonomous Area, and the Sakhalin region have developed RIS subsystems of infrastructure, innovative milieu, and framework conditions. In 2 more regions, social development of HR and infrastructure subsystems of the RIS is combined with an innovative milieu (Astrakhan region) and framework conditions (Primorye Territory). Only in 6 coastal regions of the Russian Federation the social component of innovation security is represented by the development of 1–2 RIS subsystems (one of which is usually HR): Republic of Krym, Sevastopol, Republic of Kalmykia, Krasnodar Territory, Nenets Autonomous Area, Krasnoyarsk Territory. The Republic of Daghestan got into the zone of increased risk of innovation insecurity.

The political component of innovation security was most efficiently implemented in 2015 in 44 administrative units of the Russian Federation, of which 16 are coastal (in 2016, these are 43 regions, including 12 coastal). Thus, the value of the index of the political component of innovation security in these regions is higher than the median value for all regions of the Russian Federation (for 2015 it is ≥0.206, for 2016 it is ≥0.213). In 2015, the top-10 included 4 coastal regions: Yamal-Nenets Autonomous Area, Leningrad region, Sakhalin region, Sevastopol. In 2016, top-10 regions by this indicator also included 4 coastal regions: apart from the Yamal-Nenets Autonomous Area and Sakhalin region, these are Chukotka Autonomous Area, Republic of Sakha (Yakutia).

In 2015, all 5 RIS subsystems in the context of the political component of innovative security were developed in 2 coastal regions – the Leningrad region and Murmansk region (Fig. 7). Another 6 regions have good indicators for 4 subsystems of political component of RIS with the exception of the HR subsystem for Kaliningrad region, St. Petersburg, Yamal-Nenets Autonomous Area; infrastructure element – for the Krasnoyarsk Territory; R&D – for the Arkhangelsk region; innovative milieu – for Sevastopol. In 5 regions, the political component of innovation security is represented by the development of 3 RIS subsystems, primarily HR, supplemented by R&D sector and framework conditions in the case of the Republic of Sakha (Yakutia) and the Republic of Krym; infrastructure and framework conditions in the case of the Republic of Karelia; infrastructure and R&D in the case of the Republic of Daghestan and the Astrakhan region.

The rest of the coastal regions have a poorly developed political component of innovation security, including in 2 regions being developed only in the context of 1 RIS subsystem: in the Rostov region it is an innovative milieu and the Krasnodar Territory – R&D. In 8 regions, 2 RIS subsystems are the pivot for ensuring the political component of innovation security. One of them is the framework conditions, which is supplemented in the Kamchatka Territory, the Chukotka Autonomous Area, the Magadan region, the Nenets Autonomous Area – HR subsystem; Sakhalin region and Primorye Territory –innovative milieu; and in the Khabarovsk Territory – R&D subsystem. The Republic of Kalmykia has a unique combination of developed HR and R&D subsystems in the context of indicators of the political component of innovative security.

In 2016, 6 coastal regions were able to strengthen the political component of their innovative security while 7 regions became more vulnerable (Fig. 8). The Khabarovsk Territory and Rostov region improved their political development indicators in the context of the infrastructure. Kamchatka Territory, Magadan region, Krasnodar Territory excel in innovation milieu. Chukotka Autonomous Area in R&D sector. The decrease in the index of the political component of innovation security occurred in the Nenets Autonomous Area, the Leningrad region, Kaliningrad region, the city of Sevastopol, and the most significant – in the Republic of Karelia, the Astrakhan region and the Krasnoyarsk Territory. Another 4 regions had a structural reorganization of the subindex. The RIS of the Yamal-Nenets Autonomous Area in 2016 demonstrated good indicators of political security in 4 subsystems – HR, infrastructure, R&D and framework conditions; RIS of the Arkhangelsk region on 3 subsystems – HR, R&D and framework conditions; RIS of the Murmansk region and the Republic of Krym in 3 subsystems – HR, infrastructure and framework conditions.

The geoecological component of innovation security was most efficiently implemented in 2015 in 43 administrative units of the Russian Federation, of which 13 are coastal (in 2016, these are 43 regions, including 11 coastal). Thus, the value of the index of the geoecological component of innovation security in these regions is higher than the median value for all regions of the Russian Federation (for 2015 it is ≥0.288, for 2016 it is ≥0.297). In 2015, the top-10 included 3 coastal regions: Chukotka Autonomous Area, Yamal-Nenets Autonomous Area, Leningrad region. In 2016, the top-10 included 4 coastal regions: Nenets Autonomous Area, Chukotka Autonomous Area, Leningrad region, Magadan region.

In 2015, only 1 coastal region – the Chukotka Autonomous Area, had all 5 RIS subsystems developed in the geoecological context (Fig. 9). Another 2 regions had good geoecological indicator values by 4 RIS elements: The Republic of Sakha (Yakutia) – with the exception of infrastructure subsystem, and the Rostov region – lacks the R&D sector subsystem.

In 7 regions, the geoecological component of RIS is represented by the development of 3 RIS subsystems, primarily framework conditions, supplemented by the HR subsystem (Magadan region, the Republic of Krym and Republic of Kalmykia), the infrastructure subsystem (Nenets Autonomous Area, Leningrad region, Republic of Kalmykia), the R&D subsystem (Magadan region, Yamal-Nenets Autonomous Area, Murmansk region, Nenets Autonomous Area, Republic of Krym), innovative milieu (Yamal-Nenets Autonomous Area, Murmansk region, Leningrad region, Republic of Krym). The rest of the coastal regions is poorly developed in the geoecological component of RIS, including in 12 regions in the context of 2 subsystems: R&D sector in combination with HR (Sevastopol), in combination with infrastructure (Sakhalin region), in combination with innovative milieu (Republic of Karelia, Arkhangelsk region), in combination with framework conditions (Primorye Territory, Kamchatka Territory, Krasnoyarsk Territory); HR in combination with the innovative milieu (St. Petersburg) and in combination with the infrastructure (Krasnodar Territory, Republic of Daghestan), innovative milieu in combination with the infrastructure (Arkhangelsk region) and in combination with the framework conditions (Khabarovsk Territory). The weakest geoecological component of RIS in 2015 among the coastal regions was in the Kaliningrad region.

In 2016, 5 coastal regions were able to strengthen the geoecological component of RIS; 7 regions, on the contrary, became more vulnerable (Fig. 10). The Krasnoyarsk Territory, Republic of Daghestan, and Sakhalin region improved their indicators of the geoecological development of RIS in the context of the innovation milieu, the Astrakhan region had improved HR subsystem, Kaliningrad region developed infrastructure subsystem, and Sakhalin region developed its framework conditions. The decrease in the index of the geoecological component of RIS occurred in the Magadan region, the Republic of Krym (due to indicators of the HR subsystem RIS), the Chukotka Autonomous Area and Leningrad region (due to indicators of the infrastructure subsystem), the Yamal-Nenets Autonomous Area (due to the indicators of the innovation milieu), the Rostov region and the Republic of Kalmykia (due to the indicators of the RIS framework conditions). The Arkhangelsk region underwent a restructuring in the internal composition of the geoecological subindex.

## 2 Experimental design, materials and methods

The data covers a sample of 85 regions of the Russian Federation, coverage period is 2015–2016. These macroeconomic data are collected from several reliable sources, such as the Federal Service of State Statistics of the Russian Federation (Rosstat), Scopus database, SciVal, Scientific Research Institute – Federal Research Centre for Projects Evaluation and Consulting Services (SRI FRCEC), Single portal of the budget system of the Russian Federation (Electronic budget), Scientific and technological infrastructure of the Russian Federation – Centers for collective use of scientific equipment and unique scientific installations (Ministry of Education and Science of the Russian Federation), Association of Accelerators and Business Incubators of Russia, Information and communication support system for young innovators (ICS). When building a database, comparability of indicators by size units is ensured. Standard data extrapolation method is performed whenever possible to construct a complete data series in case of missing values.

The evaluation algorithm includes 6 stages:

1) collection and analysis of indicators for selected groups, followed by their measurement;
2) formation of a statistical base of indicators for each of the regions of the Russian Federation for 2015 and 2016 based on official data;
3) normalization of indicators by the linear scaling method in order to bring all the calculated values to a single scale in the interval [0; 1], where 0 is the minimum and 1 is the maximum value of the attribute. The following formula is applied for normalization of the raw data for indicators characterizing a positive attribute:

(1) $$Z_{ij}=\frac{{\text{a}}_{\text{ij}}-{\text{a}}_{\text{j}}^{\text{min}}}{{\text{a}}_{\text{j}}^{\text{max}}-{\text{a}}_{\text{j}}^{\text{min}}},\;\text{given}\;\text{that}{\text{ a}}_{\text{j}}^{\text{max}}\neq {\text{a}}_{\text{j}}^{\text{min}}\;$$

with Z_ij_ – normalized j-value for i-region;

${\text{a}}_{\text{ij}}$ – the value of the j-index of the i-region;

${\text{a}}_{\text{j}}^{\text{max}}$ – maximum value of j-index;

${\text{a}}_{\text{j}}^{\text{min}}$ – minimum value of j-index.

The following formula is applied for normalization of the raw data for indicators characterizing a negative attribute:

(2) $$Z_{ij}=1-\frac{{\text{a}}_{\text{ij}}-{\text{a}}_{\text{j}}^{\text{min}}}{{\text{a}}_{\text{j}}^{\text{max}}-{\text{a}}_{\text{j}}^{\text{min}}},\;\text{given}\;\text{that}\;{\text{a}}_{\text{j}}^{\text{max}}\neq {\text{a}}_{\text{j}}^{\text{min}}$$

The following indicators are considered to be of a negative attribute:

• degree of depreciation of fixed assets in a full range of organizations;
• proportion of the population not using the Internet for security reasons;
• share of the total number employed in manufacturing industries working in unhealthy and hazardous conditions;
• payment for excess emissions of pollutants per organization;
• concentration of pollutant emissions from stationary and mobile sources.
4) calculation of integral indices for each cell of the matrix by the arithmetic mean method;

(3) $$\bar{{\text{Z}}_{\text{ij}}}=\;\frac{\sum_{\text{j}=1}^{\text{n}}{\text{Z}}_{\text{ij}}}{\text{n}},$$

with $\bar{{\text{Z}}_{\text{ij}}}$ – the value of the integral index for the matrix cell;

Z_ij –_ normalized j-value for i-region;

n – number of indicators in the subgroup (in this case n = 2).

5) calculation of structural indices in rows and columns of the matrix as arithmetic means of integral indices (3);
6) calculation of the final (closing) total index of innovation security level as the arithmetic mean of the structural indexes of rows and columns of the innovation security matrix of a region (Fig. 11). Furthermore, the level of innovation security of coastal regions is analyzed separately in comparison with the inland regions.

Firstly, there are indicators of the economic component of the regional innovation security, which made it possible to assess the security status of a region in 5 aspects (Table 1):

• Human resources: I1, I2 – the level of investment of economic entities in people – holders of implicit knowledge, the effectiveness of the use of human potential;
• Infrastructure: I3, I4 – the conditions for creating an information environment and establishing inter-organizational interactions with the subsequent exchange of explicit and implicit knowledge through personal and/or remote contacts;
• Research and development: I5, I6 – the level of interaction between the scientific and entrepreneurial sectors (awareness of scientists and researchers about the real problems of business entities, the relevance of the results of intellectual activity in the economy);
• Innovative milieu: I7, I8 – inclusion of enterprises and organizations in the innovation system of the region;
• Framework conditions: I9, I10 – favorability of regional environment for conducting high-risk innovation activities.

Secondly, there are indicators of the scientific and technological component of the regional innovation security, which made it possible to assess the state of RIS security by 5 aspects (Table 2):

• Human resources: I11, I12 – the quality of human resources in the field of research and development, the demand of the economy in the labor resources of the corresponding profile;
• Infrastructure: I13, I14 – conditions for creating innovation through the involvement of scientists and researchers in entrepreneurial activities;
• Research and development: I15, I16 – competitive potential of the regional research and development sector;
• Innovative milieu: I17, I18 – the level of technology transfers, the degree of technological self-sufficiency;
• Framework conditions: I19, I20 – distribution of new knowledge, including of a tangible nature in the form of scientific articles, new equipment, and technology.

Indicators of the social component of the regional innovation security, which made it possible to assess the state of RIS security by 5 aspects (Table 3):

• Human resources: I21, I22 – the quality of human resources and the potential of their reproduction;
• Infrastructure: I23, I24 – conditions for the formation of exchange knowledge flows and information exchange between the actors;
• Research and development: I25, I26 – the growth rate of new scientific knowledge;
• Innovative milieu: I27, I28 - the receptivity of the population to novelties, innovative culture;
• Framework conditions: I29, I30 – favorable conditions for inflow or/and reduction of outflow of qualified specialists to or/and from the region.

Indicators of the political component of the innovation security of a region, which allowed to assess the state of RIS security of a region in 5 aspects (Table 4):

• Human resources: I31, I32 – the degree of interaction between the authorities and the educational sector;
• Infrastructure: I33, I34 – the level of implementation of new technologies and innovations in the public sector with a consequent increase in the efficiency and transparency of its functioning;
• Research and development: I35, I36 – interest of public authorities in the results of intellectual activity of research organizations in the region;
• Innovative milieu: I37, I38 – conditions for creating an atmosphere of innovative entrepreneurship;
• Framework conditions: I39, I40 – institutional framework for the socio-economic and innovative development of the region.

Indicators of the geoecological of the innovation security of a region, which allowed to assess the state of RIS security of a region in 5 aspects (Table 5):

• Human resources: I41, I42 – localization of labor resources, environmental and technological safety of working conditions;
• Infrastructure: I43, I44 – the level of modernization of the production infrastructure, the development of environmental services;
• Research and development: I45, I46 – the level of creation of new knowledge in the field of environmental protection and environmental management;
• Innovative milieu: I47, I48 – the presence of a culture for ecological innovation;
• Framework conditions: I49, I50 – environmental quality and potential for improvement.
